# Clinical physicists’ perceptions of weekly chart checks and the potential role for automated image review assessed by structured interviews

**DOI:** 10.1002/acm2.14313

**Published:** 2024-04-22

**Authors:** Rachel Petragallo, Dishane C. Luximon, Jack Neylon, Naomi S. Bardach, Timothy Ritter, James M. Lamb

**Affiliations:** ^1^ Department of Radiation Oncology University of California Los Angeles California USA; ^2^ Department of Pediatrics University of California San Francisco California USA; ^3^ Department of Radiation Oncology Virginia Commonwealth University Richmond Virginia USA

**Keywords:** automated IGRT image review, thematic analysis, weekly chart checks

## Abstract

**Background:**

This study utilizes interviews of clinical medical physicists to investigate self‐reported shortcomings of the current weekly chart check workflow and opportunities for improvement.

**Methods:**

Nineteen medical physicists were recruited for a 30‐minute semi‐structured interview, with a particular focus placed on image review and the use of automated tools for image review in weekly checks. Survey‐type questions were used to gather quantitative information about chart check practices and importance placed on reducing chart check workloads versus increasing chart check effectiveness. Open‐ended questions were used to probe respondents about their current weekly chart check workflow, opinions of the value of weekly chart checks and perceived shortcomings, and barriers and facilitators to the implementation of automated chart check tools. Thematic analysis was used to develop common themes across the interviews.

**Results:**

Physicists ranked highly the value of reducing the time spent on weekly chart checks (average 6.3 on a scale from 1 to 10), but placed more value on increasing the effectiveness of checks with an average of 9.2 on a 1–10 scale. Four major themes were identified: (1) weekly chart checks need to adapt to an electronic record‐and‐verify chart environment, (2) physicists could add value to patient care by analyzing images without duplicating the work done by physicians, (3) greater support for trending analysis is needed in weekly checks, and (4) automation has the potential to increase the value of physics checks.

**Conclusion:**

This study identified several key shortcomings of the current weekly chart check process from the perspective of the clinical medical physicist. Our results show strong support for automating components of the weekly check workflow in order to allow for more effective checks that emphasize follow‐up, trending, failure modes and effects analysis, and allow time to be spent on other higher value tasks that improve patient safety.

## 1 INTRODUCTION

Physics weekly chart checks are an essential part of the professional responsibility of medical physicists, and are a key guardrail for identifying errors and improving quality.^1^ They are imperfect, however—the effectiveness of weekly chart checks is limited, with the sensitivity of detecting errors during physics chart checks reported in the literature ranging from 43% to 63%.^1^, ^2^ In order to examine potential approaches for improving the effectiveness of weekly chart checks, it is of the utmost importance to hear directly from medical physicists. From the field of implementation science, we know that hearing directly from the end user is crucial to improving any clinical process. In this work, we present a novel thematic analysis of the current medical physics weekly chart check process in order to identify the shortcomings of the practice. We use a thematic analysis approach to identify common themes among semi‐structured interviews of clinical medical physicists who are currently involved in their institution's chart check process.

Automation of specific physics checks has been suggested as one potential avenue for reducing errors.^3^ The earliest report of an automatic error detector dates back to 2007, with the publication of a clustering algorithm to detect plan outliers.^4^ Since then, research into automating many parts of the physics check practice can be found in the literature.^5^, ^6^, ^7^, ^8^, ^9^ These publications focus primarily on the verification of technical details or data transfer—quantitative values which are good initial candidates for automation. Such studies make the case that automating repetitive tasks frees up time for the medical physicist to investigate events or complex issues, or to spend greater time on plan quality evaluation—in sum, to devote greater cognitive effort to complicated patient cases rather than the routine checking of treatment parameters. However, limited research has been done into the applications of automation to the specific task of image guided radiotherapy (IGRT) image review. Though there is wide practice variation in whether physicists perform image review, for those clinical medical physicists who routinely review images as part of their chart checks, it can be a time‐consuming process. Thus, IGRT image review may be a good candidate for automation research.

Our work stands in light of the recent efforts of the American Association of Physicists in Medicine Task Group 275 (AAPM TG‐275) who sought to “provide practical, evidence‐based recommendations on physics plan and chart review for radiation therapy.” TG‐275′s recommendations were given following a Failure Mode and Effects Analysis (FMEA)^10^ based partly on a survey distributed to clinical medical physicists working in radiation oncology. The results of the survey deployed as part of this Task Group highlight a wide range of different chart checking practices currently being used by clinical medical physicists.^11^ While this survey covered a broad range of demographic and chart checking topics, it used multiple choice questions to capture data, which limits the potential for a deeper qualitative analysis into why such variations among institutions and professionals exists. The work we present here complements the efforts of TG‐275, in that we utilized semi‐structured interviews to understand in greater detail the current shortcomings of the weekly chart check process and examine potential ways for improving the process.

Building off the findings in TG‐275, the authors of Medical Physics Practice Guideline 11.a (MPPG‐11.a) strove to develop a professional guideline of the minimum standard of patient chart checks that should be performed in order to ensure patient safety.^12^ Both AAPM TG‐275 and MPPG‐11.a acknowledge that as new technologies, particularly new automated technologies, enter the market, the workflow of chart checks will change. Deficiencies present in the error‐detection potential of current chart checks^1^, ^2^ further justify the need for continuous improvement of the chart check process, especially as patient treatments grow ever more complex. Indeed, the authors of AAPM TG‐275 opined: “Taken together, the results of these studies indicate a need to improve plan/ chart review processes. *Improvements are needed not only in the content of what is checked but also in the implementation of these checks to improve performance through various methods including standardization and automation”* (emphasis added).

Thus, the primary goal of this work is to understand clinical medical physicists’ perspectives regarding current weekly chart check practices and identify the shortcomings in the workflow. The secondary goal is to collect feedback from clinical medical physicists to explore avenues for future work on development of automated tools to aid in the time‐consuming IGRT image review portion of weekly chart checks—an area which has been understudied in the research to date. Specifically, our group aims to understand what features of automated tools designed to assist with IGRT image review tasks would be most useful to the end user—in this case, clinical medical physicists. A critical component of this secondary analysis involves investigating any potential barriers to implementation that may arise as a clinic moves forward with adopting such technologies into their clinical workflow. Identification of barriers and facilitators is essential to maximizing the adoption and utility of a new tool, technique, or innovation.^13^, ^14^ To meet these goals, we conducted a qualitative study, using semi‐structured interviews with practicing medical physicists, and analyzed them using thematic analysis.

## 2 METHODS AND MATERIALS

### 2.1 Recruitment of subjects

Our sampling frame included clinical medical physicists who participate in their institution's weekly chart check process in both academic and non‐academic (including governmental and community clinic) centers, across a multi‐state sample including all regions of the United States. Table 1 shows how many physicists we interviewed belonging to each group. Interviewees were recruited in accordance with the approved Institutional Review Board protocol at the lead author's institution. Participants were identified through professional contacts of the authors, and no one was recruited for an interview with whom there was any supervisorial relationship with any of the authors. Nineteen semi‐structured interviews with physicists at 16 different clinics were conducted via Zoom with an approximate length of 30 minutes each. All interviews were recorded and saved for later analysis.

**TABLE 1:** Employment demographics of our interviewees.

Place of employment	Number of interviewees
Academic medical center	10
Non‐academic medical center	9

### 2.2 Quantitative survey questions

Our interview script included several quantitative survey questions. Respondents were first asked to describe their current weekly chart check workflow. They were asked how many weekly chart checks they perform per week, how often they perform IGRT image review as part of their regular weekly chart check, and how long they typically spend on this image review. They were then asked about any tools they currently use to automate any part of their weekly chart check. Physicists were asked to rate on a scale from 1 to 10, where 1 was least important and 10 was most important, the importance of (1) reducing the time spent on image review, and (2) increasing the effectiveness of image review.

### 2.3 Semi‐structured interview design

Semi‐structured interviews are a well‐established technique for data collection in various healthcare fields.^15^, ^16^, ^17^, ^18^, ^19^ Interviews follow a general script, while also allowing room for deviation from the script, potential probing questions, and for conversational flow. Our interview script included several topics. Respondents were asked open‐ended questions regarding what they view as the shortcomings of the current chart check process, focusing specifically on the IGRT image review component. Interviewees were then asked about what features of an automated tool to assist with the IGRT image review portion of chart checks they would find useful. A beta version of an automated IGRT image review tool developed by our group^20^ was shown to interviewees and feedback was collected on desired features of such an automated tool. The software interface shown to interview participants displayed an image alignment score along with previous scores for the same patient and cumulative data for the clinic overall. Finally, respondents were asked to describe the potential barriers and facilitators to use of automation in the weekly chart check workflow that they could anticipate arising in their own clinic. The full interview script, including both the survey questions and the semi‐structured topic questions, can be found in Supplementary Document A.

### 2.4 Transcription of interviews

Zoom audio recordings of the interviews were transcribed using NVivo^21^ transcription software (Lumivero, Denver, CO). Manual review and correction were performed to ensure transcriptions were accurate.

### 2.5 Thematic analysis

Thematic analysis is, broadly speaking, “a method for identifying, analyzing, and reporting patterns (themes) within data”.^22^ It has previously been identified as a research method that has broad applicability to a range of qualitative health research questions.^23^ Thematic analysis involves the collection of data, often via semi‐structured interviews or focus groups, and the subsequent analysis of common themes across that dataset. This technique is well‐established in qualitative research, with applications ranging from analyzing the perceptions of corruption in the construction industry^24^ and identifying health themes in the realm of smart home technology,^25^ to the more narrowly medical field‐focused applications of generating themes describing the views of postnatal health care^26^ and, most recently, identifying themes related to the experiences of both frontline healthcare providers^27^ and patients^28^ during the COVID‐19 pandemic. Thematic analysis offers an accessible approach to qualitative research in general,^29^ as it does not require the use of a pre‐existing theoretical framework (unlike various other approaches to qualitative research). It is well‐suited for our task of analyzing how clinical medical physicists currently conduct weekly chart checks and their feedback regarding the introduction of automation to the process.

**FIGURE 1:**
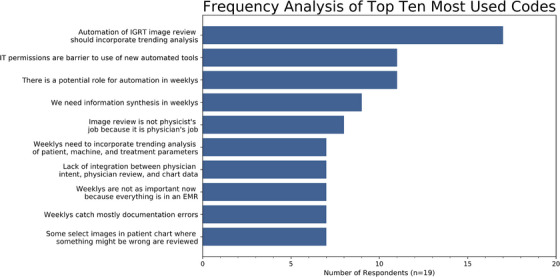
A frequency analysis of the 10 most commonly used codes in the thematic analysis.

Clarke, Braun, and Hayfield, in their book chapter on the topic,^29^ describe the six steps necessary to conduct a high quality thematic analysis: data familiarization, coding, generating themes, reviewing themes, defining and naming themes, and writing the report. We conducted data familiarization through a preliminary read‐through of our collected data, to gain a general familiarity with the type of data involved. During the coding process, we analyzed the data in finer detail and defined excerpts of text into various codes. Figure 1 depicts a frequency analysis of the 10 most commonly used codes in this step. This step remained fluid, and we utilized a mix of semantic and latent coding.^30^ Semantic codes focused on the things explicitly stated by participants, while latent codes focused more on the underlying meanings of what was said.^29^ For this study we took an inductive approach to coding, where the codes were generated based directly on the data itself, rather than a deductive approach that brought in preconceived notions about what the data might show. This approach is favored for cases in which there are no previous studies on the topic that may inform the researcher about what to expect in the data.^31^ During theme generation, we combined multiple codes into larger overarching themes that told a story about the data. In this step it was vital to not confuse themes with topics—a common issue in thematic analysis research identified by Braun and Clarke.^32^ Themes are patterns of shared meaning, characterized by a central concept. Shared topics, such as all the responses to the same interview question, do not by default fit into this narrow definition. The themes were then reviewed, and particular attention paid to thinking critically about whether the story told through the candidate themes answered our original research questions.^33^ The steps to this point were iterative, and we continually checked our codes and themes against the data to ensure that we both accurately portrayed the data through our themes and addressed the outlined goals of this work. Following multiple iterations, we defined and named our final themes, which are reported in the Results section that follows. This manuscript represents our work in writing up our findings to tell a cohesive story about the interview data through the distinct but related final themes.

The entire research team met and discussed the interviews in order to gain a general familiarity with the topics brought up by the respondents. The two coders (R.P. and J.M.L.) coded interviews independently, meeting regularly to discuss areas of agreement and of deviation in the coded interviews. Once nine interviews had been coded and reviewed, we determined that interviews were being coded the same by the two researchers and R.P. coded the remaining 10 interviews independently. New codes were still brought to the larger research team for discussion as they were developed. All members of the research team provided regular input as the codes (and later, themes) were developed and modified. The interdisciplinary nature of our team allowed us to maintain methodological rigor. Clinical investigators’ backgrounds included medical physics graduate students (R.P. and D.C.L.), clinical medical physicists (J.N., T.R., and J.M.L.), and a physician with qualitative research training and experience (N.S.B.).

## 3 RESULTS

**FIGURE 2:**
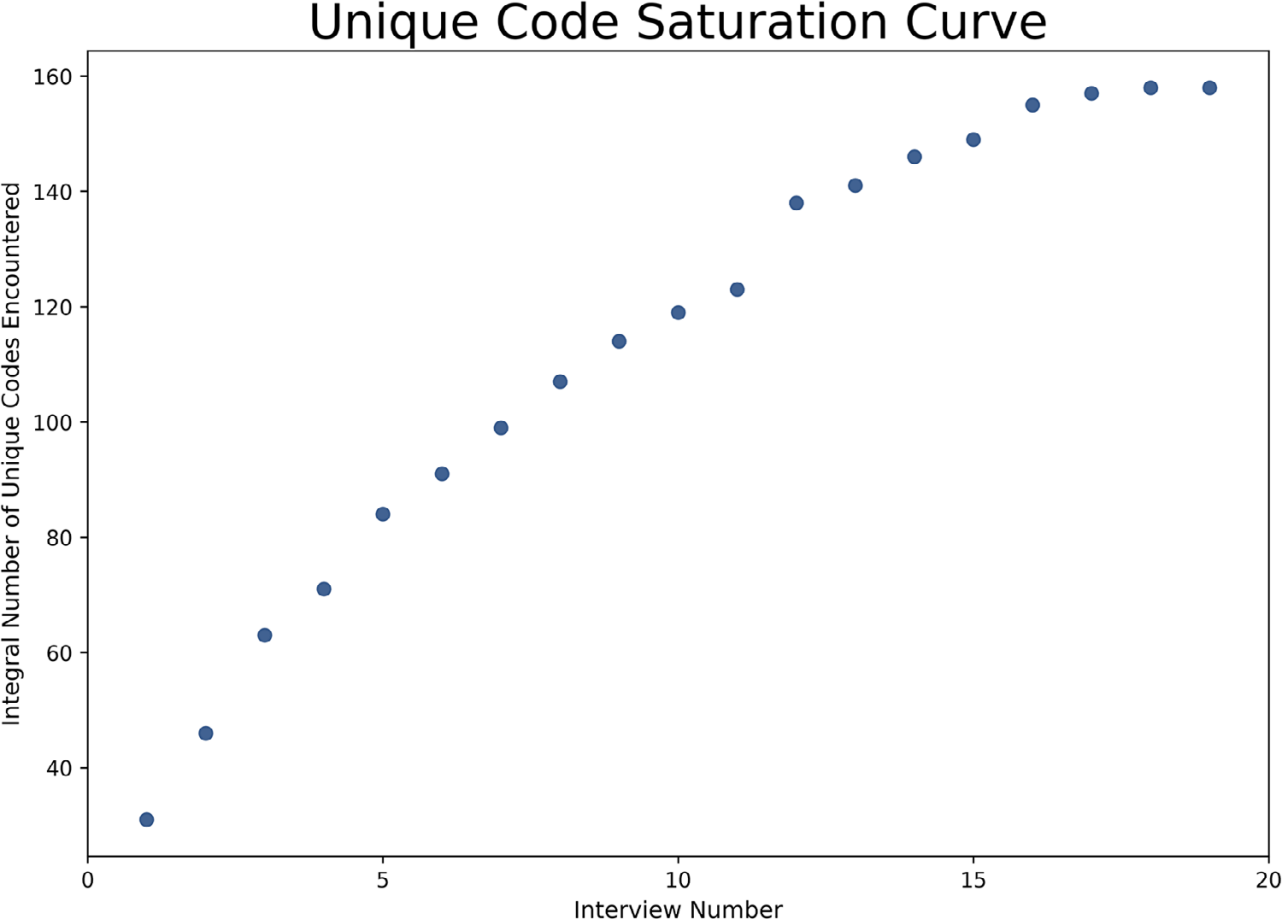
The integral number of unique codes encountered during our coding process (with 158 total unique codes identified) as a function of the interview number. From this curve, we concluded that the addition of further interviews would likely not significantly change our final themes.

We interviewed medical physicists from 16 unique institutions, located in 15 different states. Interviewees represented all geographic regions of the United States, including the Northeast, Southeast, Midwest, Southwest, and West. The saturation curve shown below in Figure 2 shows the integral number of new codes encountered as a function of interview number. Guest et al. used a similar method to show that thematic saturation in their dataset occurred within 12 interviews.^34^ Based on this curve, we determined that conducting further interviews would likely not yield a significant number of new codes and thus would likely not change our final themes.

The medical physicists we interviewed reported reviewing an average of 21 ± 7 patient charts per week (range: 8–40). 13/19 physicists reported that they do not routinely review patient setup images during their weekly check, with only 5/19 reporting that they review all images as part of their physics check. The most commonly cited reason for not reviewing images was the physicist reporting that they consider this aspect of chart checks to be the physician's responsibility, with nine respondents explicitly voicing this sentiment. While medical physicists who receive their graduate education at CAMPEP‐accredited institutions are required to receive basic training in anatomy and physiology, the guidelines offered in AAPM Report No. 365^35^ are not comparable to the intense anatomy training required of physicians. When asked how much time they spend reviewing an image in a patient's chart, the responses ranged from 30 seconds to 10 minutes (median: 2.5 minutes). The responses to this question included physicists who reported reviewing all images as part of their weekly chart checks along with those who reported reviewing only select images. For those who reported reviewing only select patient images, the majority voiced that the images they do review tend to be those that appear to have an issue or that are complicated cases, and thus significantly more time must be devoted to image review. The time difference in IGRT image review between these two groups is reflected in the large time range reported above. Thirteen physicists reported currently using some form of automation in their weekly chart check workflow, including both commercially‐available tools and in‐house software. Physicists did rank highly the value of using automation to reduce the time spent on weekly chart checks (average 6.3 on a scale from 1 to 10), but they placed significantly more value on increasing the effectiveness of weekly chart checks (that is, catching more errors), reporting an average of 9.2 on this same scale.

Thematic analysis identified four major themes across the semi‐structured interviews that we conducted, shown in Table 2.

**TABLE 2:** Four major themes developed through thematic analysis of the interviews.

Themes
1. Weekly chart checks need to adapt to an electronic record‐and‐verify chart environment
2. Physicists have the potential to add value to patient care by analyzing images without duplicating the work done by physicians
3. Greater support for trending analysis would increase the value of weekly checks
4. Increased automation has the potential to make weekly checks a higher value activity

1 **Weekly chart checks need to adapt to an electronic record‐and‐verify chart environment**

While physicists we interviewed shared that they personally had caught errors during the course of their weekly chart checks, these errors tended to be documentation errors. Physicists expressed frustration with the current weekly chart check workflow, specifically calling out the long list of documentation checks as an inefficient use of time.

*“I would say the vast majority are small things like documentation, like something was left out or like a document wasn't put in or, you know, maybe something wasn't entered right.”* (Non‐academic medical center)

*“Here's what I'm going to hear that I've made a suboptimal chart check, it's because something wasn't billed correctly. Some document that is there wasn't approved. Or some document that was there was mislabeled…which has nothing to do with actually checking if the treatment went well or not.”* (Academic medical center)

Physicists called attention to the fact that many of the things that are still being checked during the physics weekly chart checks are holdovers from an earlier era of paper charts. Specifically, physicists mentioned many failure modes are now extremely improbable with the electronic record‐and‐verify systems in place in most clinics, and yet these failure modes are still a major part of the weekly checks.

*“I think nowadays it's just, there's so many things that are like more robust, more automated. There's just so much less room. You're not going to have the wrong MU, like the machine is not going to let you have the wrong MU. So things like that that could have been a big problem before are not really issues anymore, and we're still treating them like they are.”* (Non‐academic medical center)

*“We used to check SSDs and some machines we have, like with Siemens, like surface mapping. So guess what? The SSDs are always right.”* (Academic medical center)

*“We're looking at the paper, we're calculating the number of fractions, the dose and total dose. We're doing all this manually in the past, as you remember. Now like in the Varian system, everything is calculated for you.”* (Non‐academic medical center)

Instead, physicists said that a greater focus and priority should be given to directing cognitive resources and expertise towards investigating anomalies in patient charts, and away from menial chart check tasks.

*“There should be some way to filter out things so that you pay attention to the [chart] that's important. Most of them go like clockwork. But there are those odd ones where you have to stop and think, and it would be better to devote time to those.”* (Academic medical center)

*“And so effectively, your weekly chart checks would just be like reviewing anything that looks unusual.”* (Non‐academic medical center)

Overall, physicists felt that current weekly chart check workflows were inefficient and of low value:

*“Spending five or ten minutes on the patient just for the sake of doing that, which seems to be kind of more the approach right now, I don't think is really useful.”* (Academic medical center)

*“It seems like a lot of time that goes into something that doesn't add a whole lot of value. And I personally, I question if that's where we should be spending our time.”* (Non‐academic medical center)

2 **Physicists have the potential to add value to patient care by analyzing images without duplicating the work done by physicians**

The majority of the physicists we interviewed reported that they don't routinely review patient setup images. The most commonly given reason for this was that the physicist views IGRT image review as a physician responsibility.

*“I kind of leave that to the physician and look for their feedback, if there's a problem with the images.”* (Academic medical center)

*“We just take a glance at them then make sure they are reviewed but we're not necessarily the ones review[ing] them. It's the physicians’ charge code, so it's physicians’ responsibility.”* (Academic medical center)

An additional factor limiting physicist review of images is a perception that they are not appropriately trained to do so:

*“Some of us don't even know that much of anatomy to say, you know, whether the image is good or not good. That's how I see it from my point of view, right? I'm not clinically trained in looking at anatomy and telling what's right, what's wrong.”* (Non‐academic medical center)

*“I think a physician probably cares more about details of the anatomy alignment. I'm looking at the rough error there.”* (Non‐academic medical center)

Several interviewees reported that their interpretation of setup images could be hindered by the fact that the physician alignment priorities were not documented in a way that was easy to integrate into the weekly chart check process. Thus, for those physicists who did report reviewing images regularly or for the patient cases where a physicist image review was necessary, a significant amount of time was spent on understanding setup instructions rather than on a review of the images themselves.

*“If there's an issue with the setup, I have questions like, why does this setup look this way? There's not really a good communication between what was done at the machine versus what someone can look up later to understand the decisions they made at the time of treatment.”* (Academic medical center)

*“I think probably the thing that takes me the longest is trying to figure out what the physician has ordered.”* (Academic medical center)

However, many physicists still expressed that IGRT image review was one area where their technical expertise could potentially have the greatest impact on patient care and treatment quality.

*“That's probably the most useful thing you can do is like, look at, verify images.”* (Non‐academic medical center)

*“So I think that the place we have the most room to gain … or to add the most value is a better review of our images. But the way we do it right now, a human looking at it and then having to remember what they looked at last week or from two days, like the Monday versus the Tuesday image, is really ineffective.”* (Academic medical center)

When IGRT image review is being done by a physicist, it's currently done in isolation. Interviewees expressed that opportunities likely exist to utilize automation for a different look at image review. For example, automation could allow physicists to analyze large volumes of image data quantitatively or to look at setup images as a continuation of trending image alignment metrics. The introduction of a new algorithmic approach to image review and the interpretation of its results would be well within the purview of the clinical medical physicist, and could add a new layer of information not currently accessible, ultimately improving patient safety.

*“If you have to do [image review], it can take a ton of time, and you're still left guessing at the end whether it was right or wrong. So something that's objective, that can look through a large volume of data very quickly, that would be helpful.”* (Academic medical center)

*“One of the biggest issues is there's no way to assess qualitatively; you look at each one in a vacuum.”* (Academic medical center)

3 **Greater support for trending analysis would increase the value of weekly checks**

A lack of trending information was identified as a major shortcoming in the current weekly chart check process. Interviewees specifically reported that the large amount of data was difficult for a human to interpret without looking at it as part of a larger trend, but that such trending information is difficult to access in the current weekly chart check workflow.

*“I believe that there is more value to trending different parameters from the Linac and from the imaging as you do it and from the delivery basically all together than to the actual review of the chart because everything is computerized.”* (Non‐academic medical center)

*“We have no way to trend right now in ARIA. You know, there's no automated report to say, show me all the head and neck patients that have large target volumes and their daily shifts or their daily image alignment score, right?”* (Academic medical center)

*“That's something that computers and the software can do really well and humans can't. With all this AI stuff—like humans, we can't trend well. But computers can, and they can get that information in a way that's useful for us.”* (Academic medical center)

The importance of trending was highlighted even further when interviewees talked about image review. Physicists expressed the need for a greater focus to be given to trending analysis in IGRT image review, citing bladder and rectal filling in prostate cases, weight loss and tumor shrinkage in head and neck cases, and adaptive re‐planning as examples of patient cases where tracking and trending setup images over time could help them be more efficient and proactive in their chart checks.

*“I, as a human, have a hard time integrating the information from one daily image to another. And I think there's a lot of information to be gained there.”* (Academic medical center)

*“I do not know exactly how much the structure of the organs have changed. I don't have like any trending of, as I said, bladder filling or rectal filling for prostates. I don't have any tracking in terms of volume changes of the patient's GTV in a head and neck case.”* (Non‐academic medical center)

*“One issue that I have is really if you're considering re‐simulating a patient due to anatomic changes, that's a very, you know, qualitative decision at this point. It would be nice to have some sort of algorithmic approach to this with some threshold.”* (Non‐academic medical center)

4 **Increased automation has the potential to make weekly checks a higher value activity**

Physicists expressed that many of the things currently being checked on their weekly chart checks are documentation or numerical checks, which are prime candidates for a transition to automated checks. Opportunities likely exist to utilize automation for many of these checks and rethink what should be manually reviewed by the physicist as part of their weekly chart check process.

*“I mean, we lack a lot of automation. I think there's a lot of things that can be automated, as you say, a pre‐check, weekly check and all kinds of stuff can be automated.”* (Non‐academic medical center)

*“Because we lack automation to really just kind of turn over every stone [chart checks] are, you know, a medium level of activity.”* (Academic medical center)

*“More of that kind of stuff is better and then, you know, it's safer and it takes away all the bean counting of our job, which is great.”* (Non‐academic medical center)

One benefit of automation is that humans have a limited attention span when faced with repetitive tasks, and so might not catch all the errors present in the data. Physicists expressed this concern directly, citing that human err likely makes weekly chart checks less effective than they should be.

*“I think it's better to catch those things than a human eye. And because you are doing that as a routine, you get fatigued and you skip things.”* (Non‐academic medical center)

*“Most of the time, everything works like clockwork, so you become accustomed to that. But then there are those that for some reason don't fit and you have to have either a sharp eye or just be very lucky to spot it.”* (Academic medical center)

Many of the physicists we interviewed expressed that a thorough weekly chart check is very effective at catching egregious errors, but it is not scalable because of the time it takes. In their view, the current weekly chart check process contains many inefficiencies and ultimately takes too much time and mental energy for every single patient check to be done thoroughly.

*“I feel like the problem with the weekly chart checks is number over quality.”* (Academic medical center)

*“In some ways I'm impressed and some other ways I'm like, well, how much time does it take spent on a weekly check to catch that? And how much is actually luck and how much is there in parallel that we don't catch. I mean, to me, it always seems like it's the visible tip of the iceberg, and if we caught that there were probably quite a few other things.”* (Academic medical center)

Interviewees pointed out that a key opportunity for automation was the flagging of anomalies for further investigation. Such tools could allow for physicists’ time and cognitive resources to be directed in a more focused manner on investigating the complex patient cases, ultimately improving patient safety in the clinic overall.

*“An ideal setup for me for chart check would be something that is really good at flagging suspicious things.”* (Academic medical center)

*“With the machine, automation can kick in to replace that part and we spend time to investigate the real problem, that will be nice.”* (Non‐academic medical center)

*“We'll catch errors more consistently than all of us doing slightly different weekly chart check. Second we'll be efficient. And so we free up time for us to do something more constructive than repetitive work.”* (Academic medical center)

**FIGURE 3:**
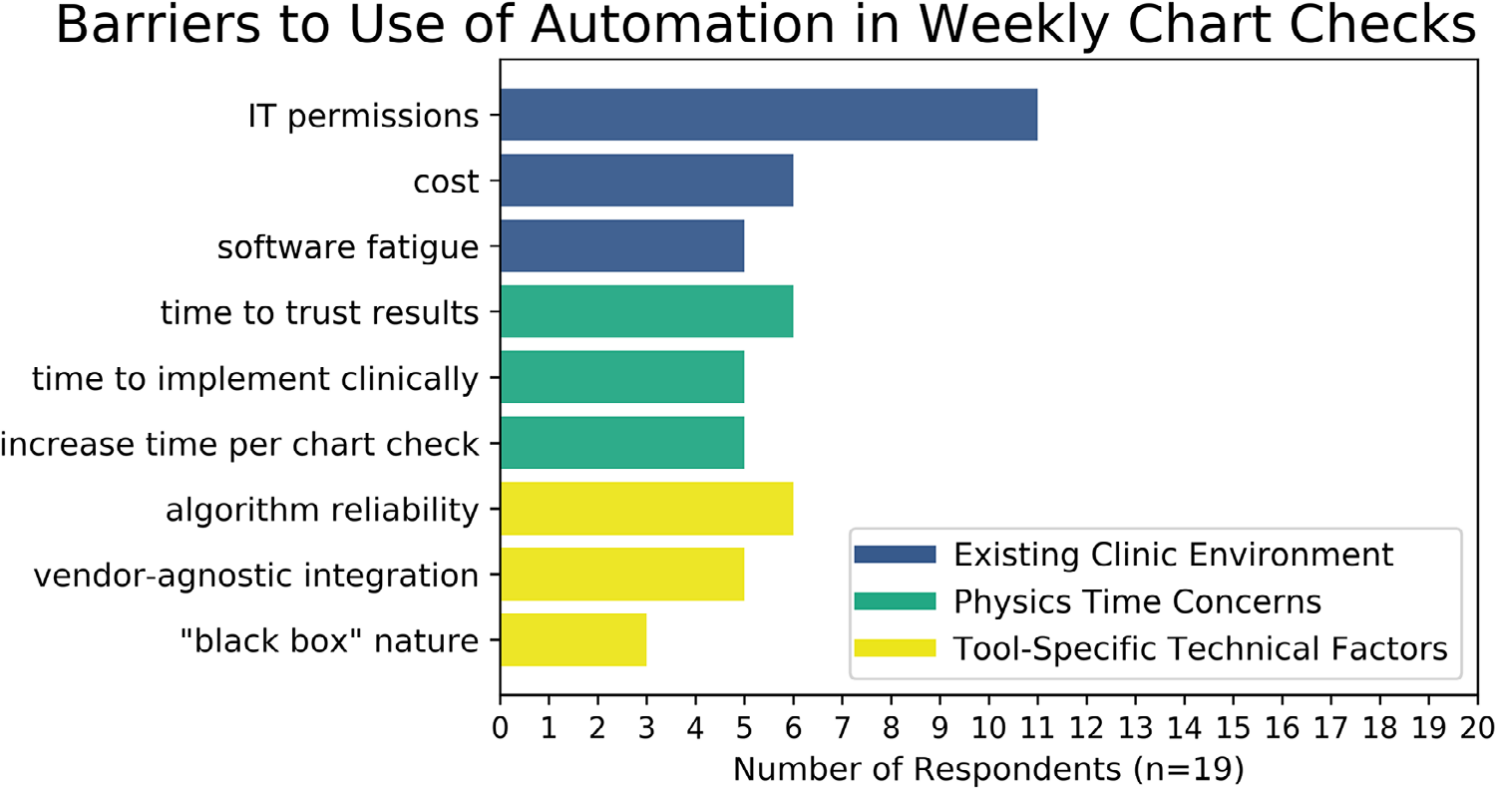
The most commonly reported barriers to the use of automation in the weekly chart check workflow.

Finally, three broad categories of potential barriers to use of automation in the weekly chart check workflow were reported in response to our semi‐structured interview questions on the topic: existing clinic environment, workload and time concerns, and tool‐specific technical factors. The most commonly reported barriers to use in each of these categories are shown in Figure 3. The single most commonly identified barrier to clinical use of an automated tool was overcoming their clinic's IT permissions, with 11 out of the 19 respondents reporting this barrier could hinder their adoption of such tools. Other existing clinical environment factors that could be barriers to implementation included the cost of a new tool and software fatigue (meaning a wide variety of software is already in use in their clinic, and there is low motivation to introduce more). A second area of concern for physicists was that their clinic's adoption of automated tools could, perversely, lead to increased workload on the physics staff. Physicists reported concern that the time needed to trust algorithm results, the time needed to implement a new tool, and the time needed to operate the software and interpret results could all lead to increased strain on an already busy physics staff. Third, physicists pointed to several tool‐specific technical factors that could present barriers to clinical adoption. Algorithm reliability (or lack thereof), vendor‐agnostic integration, and a lack of trust in “black box” machine learning algorithms were all mentioned as potential barriers to clinical use.

## 4 DISCUSSION

Our results show that most physicists believe that change of their weekly chart check procedures is urgently needed. As technology change in the radiation oncology clinic has continued and indeed accelerated over the past few decades, weekly checks have not kept pace. Physicists pointed out many checks currently done as a matter of routine practice in their weekly chart checks that no longer add to patient safety the way they did when they were first introduced. This reflects a key recommendation of AAPM TG‐275, which points to the need for an AAPM TG‐100 FMEA approach to be taken in regards to the physics weekly chart checks. It is noteworthy that none of the physicists we interviewed directly mentioned the need for FMEA to be considered in weekly chart checks, nor was TG‐275 mentioned even indirectly. TG‐275 and the associated practice guidelines are relatively recent; perhaps it will simply take time for this recommendation to be widely adopted.

Physicists rated the value of increasing the effectiveness of weekly chart checks higher than decreasing the time spent. This sentiment could be attributed to physicists’ dedication to safety or, due to the perfunctory nature of many current chart check tasks, physicists may be spending minimal time on weekly checks to begin with. Physicists we interviewed spoke to the human error and mental fatigue aspects of weekly chart checks as limitations in their value. Clearly, physicists feel that chart checks have imperfect sensitivity to detect errors, and evidence exists in the literature to support this.^1^, ^2^, ^3^

Our data suggest that the field of medical physics could examine the potential for physicists to be more involved in image review. Not all physicists perform image review as part of their weekly chart check tasks, as we have shown in this study. It is beyond the scope of this work to set forth guidelines for the clinical medical physicist regarding image review, but the results presented here may help inform the debate of whether and how medical physicists should be involved in the process. Currently, physicists are under ever increasing time pressures,^36^, ^37^ which means that more and greater complexity chart checks are being done with less resources. Physicists have the potential to add value in a different, perhaps more algorithmic, approach to image review. The physicists we interviewed expressed the sentiment that if physicist resources could effectively be freed up for image review, this could be a valuable additional layer of safety in the clinical workflow. Weekly chart checks currently take a good deal of physicists’ time to complete, but if the routine checks could be automated or otherwise made more efficient then physicist efforts could be more realistically dedicated to analyzing new image review metrics.

Physicists we interviewed felt that there was a potential role for automation in the weekly chart check workflow to supplement the human checks, and did not express concern about automation replacing them in the clinic. Specifically, physicists reported the desire for more automated tools that flag anomalies, allowing them to analyze complex cases and investigate complex problems. AAPM TG‐275 suggests that software vendors should continue to work towards the development of automated tools that can assist with chart review tasks, while also being cognizant of the limits of automation. Automation bias is a real and well‐documented concern,^38^, ^39^ and the development of automated tools should be coupled with research into minimizing the associated automation bias. It is noteworthy that only one physicist cited job security as a barrier to use of automated tools in their weekly chart checks. This contrasts strongly with job security concerns related to automated treatment planning, which is a considerable source of worry for dosimetrists with one study finding that 21/34 dosimetrists explicitly voiced this concern.^40^ While computers and automation can assist in the process and make the weekly checks more efficient and more effective, physicists still seem to feel that there will be a place for them in the clinic. This sentiment fits neatly within one of the stated trajectories of Medical Physics 3.0 (MP3.0),^41^ namely, Sustainability, which argues for a redistribution of the medical physicist's responsibilities in order to better pursue value‐based goals. Physicists can preserve a place for themselves in the clinic by advocating for new automated tools that complement their role and allow them to shift their focus to complex tasks, thereby increasing their value to the clinic overall.

When interviewees were asked about what features of a new automated weekly chart check tool would be important, every single one of them highlighted the need for trending analysis. Many of the checks currently being performed in the weekly chart check workflow are performed in isolation, and the physicists we interviewed spoke of the value that trending analysis could add. As more and more automated tools are put forward by industry, our data suggest that examining what sort of trending analysis support can be offered by such tools could be useful to clinical medical physicists. Physics checks encompass a huge volume of data, which is potentially an opening for AI‐based dimensionality reduction techniques to assist with a more effective chart review. While automation can perhaps support a greater emphasis on trending analysis, physicists pointed out that their expertise would still be needed in the clinic to interpret such results.

Even as the applications of automated technologies continue to expand, there is still a gap between research findings and the clinical implementation of those findings. As new automated technologies are developed, a conscious effort should be made to study the barriers to use and ensure that advances in research translate to advances in clinical care. This can be of particular importance in the United States, where widespread dissemination of evidence‐based best practices is frequently hampered by the wide diversity of healthcare settings.^42^ We found that the most commonly reported barriers to use of automated weekly chart check tools can be broken down into the following categories: existing clinic environment, workload and time concerns, and tool‐specific technical factors. As researchers and developers continue to automate ever more of the physics chart check tasks, a focused effort should be given to understanding these barriers and the implications for clinical adoption of their automated tools.

Our study has a number of limitations. While we attempted to reach a diverse group of medical physicists through our recruitment efforts, we are cognizant of the limitations we faced. Only two of our 19 respondents reported employment at community clinics, although we did reach seven physicists employed at governmental health clinics. Taking these two groups together, we interviewed nine physicists employed at non‐academic medical centers in comparison with the 10 physicists employed at academic medical centers. Our respondents represented 16 unique institutions and 15 states from all regions of the country (Northeast, Southeast, Midwest, Southwest, and West), although our cohort was limited to clinical physicists currently practicing in the United States. The conclusions we reach in this study may not translate well to other countries, where the chart check requirements could be vastly different for clinical medical physicists. By soliciting participants based on professional contacts of the authors, we may have introduced a selection bias in the physicists we considered for this study, but this does not necessarily translate to a true self‐selection bias in the respondents. Of the 21 clinical physicists who were contacted about this study, only two declined to participate or did not respond. Related to selection bias, we may have interviewed physicists with strong opinions on weekly chart checks or automation research, and not reached those who are ambivalent on the topics. Finally, we acknowledge that our sample size of 19 respondents is not large. This is not atypical for a qualitative research study; studies published in recent years investigating Diversity, Equity, and Inclusion in radiation oncology^43^ and resilience among medical physics residents^44^ have conducted semi‐structured interviews with cohort sizes of 26 and 32, respectively. In addition, a rigorous thematic analysis includes depth of data collection and achieving thematic saturation, more than the specific number of people interviewed. Our structured interview script was detailed, requiring 30 min to complete, and each interview provided a wealth of information. As noted above, during the course of our thematic analysis of these 19 interviews, we concluded that thematic saturation had been achieved and that further interviews would likely not yield additional themes.

## 5 CONCLUSION

In this work, we use a novel thematic analysis to identify the shortcomings of the weekly chart check process from the perspective of the clinic medical physicist. We describe four major themes, which each complement the findings and recommendations of AAPM TG‐275. Clinical medical physicists described both a need for greater automation in the weekly check process and a sentiment that the process itself must adapt in light of increasingly automated systems. As automated technologies continue to become increasingly prevalent in the clinic, the FMEA approach advocated by TG‐275 and the value‐based care focus advocated by MP3.0 suggest that the current way of doing weekly chart checks needs to be re‐evaluated. This would allow for more effective physics chart checks that emphasize follow‐up, trending analysis, FMEA, and other higher value tasks that improve patient safety.

## AUTHOR CONTRIBUTIONS

Rachel Petragallo participated in interview design, interviewed respondents, performed the thematic analysis, and drafted the manuscript. Dishane C. Luximon provided input on the thematic analysis and edited the manuscript. Jack Neylon interviewed respondents, provided input on the thematic analysis, and edited the manuscript. Naomi S. Bardach participated in interview design, provided input on the thematic analysis, and edited the manuscript. Timothy Ritter provided input on the thematic analysis and edited the manuscript. James M. Lamb conceptualized and provided overall direction to the study, participated in interview design, interviewed respondents, and edited the manuscript.

## CONFLICT OF INTEREST STATEMENT

The authors have no relevant conflicts of interest to disclose.

## Supporting information

Supporting Information

## Data Availability

The data that support the findings of this study are available on request from the corresponding author. The data are not publicly available due to privacy or ethical restrictions.
