# Supplementary material for: Clinical physicists’ perceptions of weekly chart checks and the potential role for automated image review assessed by structured interviews
Source: J Appl Clin Med Phys. 2024 Apr 22;25(5):e14313. doi: 10.1002/acm2.14313 (PMC11087166; doi:10.1002/acm2.14313)
Supplement: Supplementary file 1 — Supporting Information [file ACM2-25-e14313-s001.docx]

We are requesting your feedback regarding a software tool being developed by the Radiation Oncology Safety, Automation and Machine Learning (RO-SAML) research group. It is hoped that this tool could help clinical radiation oncology physicists perform a validation of IGRT alignments as part of a weekly physics check. We will be asking you about your weekly chart check workflow, your experience with quality control automation, and about the proposed software tool. We estimate that this interview will require 30 minutes of your time. We sincerely thank you for your participation.

The researchers will do their best to make sure that your private information is kept confidential. A video recording of this interview will be stored for the duration of this study. All data you provide will be stored in a password-protected file on a secure network server. Following completion of the study, all identifiable data will be destroyed.

If you have any questions please contact the principal investigator: James Lamb at [JLamb@mednet.ucla.edu](mailto:JLamb@mednet.ucla.edu). If you wish to talk with someone other than the researchers, you may contact the UCLA Office of the Human Research Protection Program (OHRPP) by phone: (310) 206-2040; by email: [participants@research.ucla.edu](mailto:participants@research.ucla.edu) or by mail: Box 951406, Los Angeles, CA 90095-1406.

First I will be asking you a few questions about your current workflow:

1. How many chart checks do you perform per week?
2. How many setup/treatment images do you review per week?
   1. About how many images are generated per patient per week?
   2. Do you review kV and/or MV planar images? Do you review CBCT images?
   3. Do you review them in Offline Review, or a similar “live” system that allows review of the fusion, or do you review them in PDF form only?
3. Please talk me through how long you think you might spend reviewing the images per patient for a weekly check. Consider all time costs, including the time it takes to open the patient in the review software, load each image, and close the patient.

Prompts:

- 1. How fast does it go with images that are easy to check? What fraction of images is this?
  2. How fast does it go with images that are more subtle to check?

1. Do you use any products that automate weekly chart checking? Automate pre-treatment checks?
   1. Prompt with Radformation ChartCheck, ClearCheck, Varian Chart QA.
2. What are the shortcomings of the current weekly chart check image review process in your view?

Our group is developing software to work with the ARIA Oncology Information System from Varian. It interrogates the treatment database at the end of the day, identifies new images, and uses an AI tool to assess the quality of the alignment and flag any anomalies. A summary report then is automatically generated for all patients.

(Show Powerpoint slides here)

1. We think that the two most useful potential aspect of the tool are 1) to reduce the amount of time spent on weekly checks without increasing error rate; and 2) to increase the effectiveness of weekly checks (catch more errors). Please rate the relative importance of the following:
   1. Reduce the amount of time spent on weekly checks without increasing error rate (1-10 1 being the lowest importance and 10 being the highest)
   2. Increase the effectiveness of weekly checks (catch more errors) 1-10

Prompts/probes about why?

1. Tell us about how you feel about the value of weekly chart checks? Do weekly chart checks frequently catch errors?
2. Can you tell us an error you’ve caught with weekly chart checks? Was it correctible? Did it lead to a process change?

As we develop the tool, we are looking to understand what would be most helpful for people who are going to use it.

7. How would you prefer to interact with this tool?

- Program with a GUI that you start up when you want to use it?

- Receive an emailed report daily?

- Receive an emailed report at a configurable time interval?

- Any other method?

1. There are a number of features we are considering including in this tool. I am going to review a list of them to see whether you would find them useful or not and why.
   1. Would you like to see an alignment score for every image? Why or why not?
   2. Would you prefer that the tool flags images with alignment scores falling below a pre-determined threshold, or would you prefer for it to flag images with the lowest percentile alignment scores (so that it always flags a fixed percentage of images)?
   3. Would you like a graphical display of alignment scores? Why or why not?
   4. Would you like to see per-patient trendlines? Why or why not?
   5. Would you like the tool to include the images themselves, or would you revert to Offline Review to review flagged images? Why or why not?
   6. Are there any other features you would suggest that I have not already mentioned?
2. If you were using this tool, what might make you more comfortable decreasing the amount of time reviewing images not flagged by the tool?

We are also interested in understanding more about potential barriers to using the tool.

1. What barriers to your use of the tool might you anticipate?
   1. One potential barrier is IT permissions. If the software tool could be easily loaded onto your Varian network, how difficult do you anticipate it would be to obtain permission to install the software? (probe if difficult) What would make it difficult? (Probe if not difficult) What would make it not too difficult?
   2. Existence of appropriate computational infrastructure?
   3. Consider a recent example of technology adoption in radiation therapy that was (or was not) successful. What factors determined that technology’s success? What were the barriers to adoption that were or were not overcome?
2. Would you be interested in using this tool if it was available?
   1. (If yes) why? (If not), why not?
   2. Probe the issue of whether they review every single image or not – does this help improve safety or reduce time or both?

13. Do you have any further comments, suggestions, concerns, or ideas regarding this tool?
